# Expanding CXCR4 variant landscape in WHIM syndrome: integrating clinical and functional data for variant interpretation

**DOI:** 10.3389/fimmu.2024.1411141

**Published:** 2024-07-08

**Authors:** Katarina Zmajkovicova, Keith Nykamp, Grace Blair, Melis Yilmaz, Jolan E. Walter

**Affiliations:** ^1^ X4 Pharmaceuticals (Austria) GmbH, Vienna, Austria; ^2^ Invitae, San Francisco, CA, United States; ^3^ Division of Allergy and Immunology, Department of Medicine, Johns Hopkins All Children’s Hospital, St Petersburg, FL, United States; ^4^ Division of Allergy & Immunology, Department of Pediatrics, Morsani College of Medicine, University of South Florida, Tampa, FL, United States; ^5^ Division of Allergy and Immunology, Massachusetts General Hospital for Children, Boston, MA, United States

**Keywords:** WHIM syndrome, congenital neutropenia, primary immunodeficiency disease, CXCR4, genetic testing, functional assays

## Abstract

Warts, Hypogammaglobulinemia, Infections, Myelokathexis (WHIM) syndrome is a rare, combined immunodeficiency disease predominantly caused by gain-of-function variants in the *CXCR4* gene that typically results in truncation of the carboxyl terminus of C-X-C chemokine receptor type 4 (CXCR4) leading to impaired leukocyte egress from bone marrow to peripheral blood. Diagnosis of WHIM syndrome continues to be challenging and is often made through clinical observations and/or genetic testing. Detection of a pathogenic *CXCR4* variant in an affected individual supports the diagnosis of WHIM syndrome but relies on an appropriate annotation of disease-causing variants. Understanding the genotypic-phenotypic associations in WHIM syndrome has the potential to improve time to diagnosis and guide appropriate clinical management, resulting in a true example of precision medicine. This article provides an overview of the spectrum of *CXCR4* variants in WHIM syndrome and summarizes the various lines of clinical and functional evidence that can support interpretation of newly identified variants.

## 1 Introduction

WHIM syndrome is a rare autosomal dominant combined immunodeficiency disease (OMIM #193670). The WHIM acronym refers to a set of typical clinical features of the disease, namely warts, hypogammaglobulinemia, infections, and myelokathexis. However, the full tetrad of symptoms is found only in a minority of individuals (22%-38%) (1–3). The most penetrant clinical and laboratory phenotypes in individuals with WHIM syndrome are severe neutropenia due to impaired release of neutrophils from the bone marrow, lymphopenia, and recurrent bacterial infections (1–3). The disease was first described by Zuelzer in 1964 (4), but the genetic etiology was only discovered 39 years later when variants in the cytoplasmic C-terminal tail of C-X-C chemokine receptor 4 (CXCR4) were linked to the pathology of WHIM syndrome in seven independent kindreds (5). As per European Society for Immunodeficiencies–Pan-American Group for Immunodeficiency diagnostic criteria, identification of a CXCR4 variant in the intracellular C-terminal tail of the receptor or an activating CXCR4 variant supports the definitive diagnosis of WHIM syndrome (6). Owing to the availability of sequencing, the spectrum of *CXCR4* ^WHIM^ variants has grown dramatically since the first discovery of 3 disease-causing variants (5, 7, 8). In this review, we aim to summarize the current knowledge of the landscape of *CXCR4* variants in WHIM syndrome, as well as provide an overview of functional assays that can support interpretation of newly discovered *CXCR4* variants and their pathogenic role in the disease.

## 2 *CXCR4* variant landscape in individuals with WHIM syndrome

**Figure 1:**
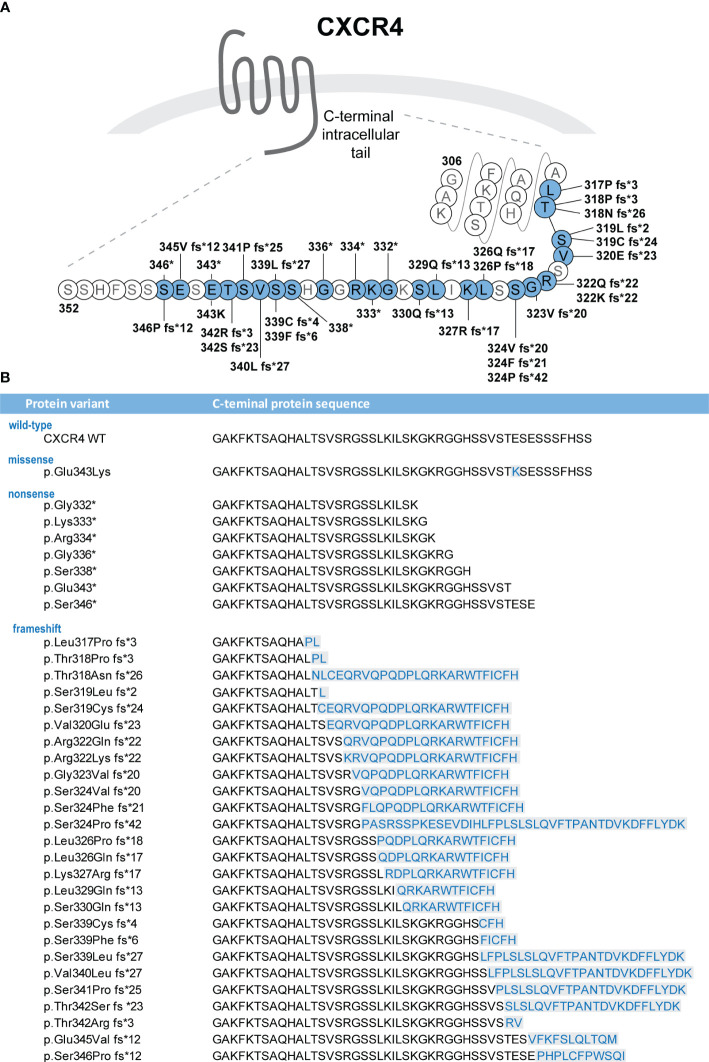
**(A)** CXCR4 variants identified in individuals with WHIM syndrome are localized in the C-terminal intracellular tail of the receptor (24). The figure indicates protein variants identified to date and positions at which they alter the wild-type sequence of the CXCR4 protein. **(B)** Protein sequences of the C-terminus variants are shown. Missense variants and *de novo* sequences resulting from the frameshift variants are highlighted in blue color. *: translation termination codon (stop codon).

Heterozygous C-terminal CXCR4 variants are found in ≈90% of individuals with clinical diagnosis of WHIM syndrome (2, 3). p.R334* (c.1000C>T), 1 of the 3 originally discovered pathogenic CXCR4 variants (5), is the most frequent one, accounting for 47% to 55% of CXCR4 variants in WHIM syndrome (1–3). p.S338* (c.1013C>G or C>A) is the second most frequent; it is found in 16% to 17% of individuals with CXCR4 variants (1–3). As of March 2023, 33 additional C-terminal variants have been reported, often occurring *de novo* in a single individual or in several members of 1 family (2, 5, 9–23) (**Figure 1A** (24), **Supplementary Table 1**). The total of 36 variants can be divided into 3 subgroups, composed of 1 missense, 8 nonsense, and 27 frameshift variants (**Supplementary Table 1**). The affected region spans amino acids (aa) 317 to 346, with nonsense variants occurring between aa 332 to 346 and frameshift variants between aa 317 and 346. Both +1 and +2 frameshift variants have been detected in individuals with WHIM syndrome (**Figure 1B**). The span of CXCR4^WHIM^ variants is very similar to somatic CXCR4 C-terminal variants found in Waldenström macroglobulinemia, a rare indolent B-cell lymphoma (25, 26). Notably, the number of distinct variants is higher in Waldenström macroglobulinemia, and the C-terminal region affected by frameshift variants extends to position T311 (p.T311Ifs*33) (26) and to K327 for nonsense variants (p.K327*) (27). It is therefore likely that additional novel variants will be identified in individuals with WHIM syndrome in the future, especially due to many possible combinations of indels leading to frameshift variants in the C-terminus of CXCR4.

## 3 *CXCR4* variant interpretation in the setting of genetic testing

Interpretation of variants’ pathogenicity for the underlying condition is an essential aspect of genetic testing. Variant classification is performed according to internationally accepted standards and relies on several lines of clinical and functional types of evidence (28, 29).

One line of clinical evidence in variant classification considers whether the detected variant has been previously observed in clinically affected individuals and families with a condition while being absent in unaffected individuals (28, 29). To access genetic variant data, clinicians and investigators frequently use reference databases such as ClinVar (30), an international public archive of variant-condition interpretations hosted by the National Center for Biotechnology Information. As of December 6, 2023, ClinVar contained entries for 19 of the 36 *CXCR4* variants that have been identified in individuals with WHIM syndrome in scientific literature, with 15 of them classified as likely pathogenic or pathogenic, 4 as variant of uncertain significance (**Supplementary Table 1**) (31). Hence, *CXCR4* variant annotation in ClinVar has been outpaced by scientific reports, and to accurately classify the newly identified *CXCR4* variants, it is critical to review the recent literature. Of note, a thorough variant interpretation per American College of Medical Genetics and Genomics – Association for Molecular Pathology (ACMG-AMP) guidelines is rarely performed in publications, with a few exceptions (2, 32) (**Supplementary Table 1**), but such publications still represent a valuable resource for correlating genotype with clinical phenotype.

For a variant to be classified as pathogenic or likely pathogenic, it should segregate with the disease or occur *de novo* in the affected members of a family, and the allele frequency should be as low as the expected prevalence of WHIM syndrome (29). As of November 29, 2023, a majority of the known *CXCR4* ^WHIM^ variants were absent in gnomAD (formerly ExAC), the largest publicly available aggregated dataset of human variant allele spectrum and frequencies (33). Two variants, c.1006G>T/p.G336*, pathogenic, and c.1013C>A/p.S338*, likely pathogenic, are each found in the gnomAD dataset at allele frequency 1.60e-6 (34).

*In silico* tools that predict the impact of sequence variants can also bring valuable input into the variant classification process (29, 35). Specifically, combined annotation‐dependent depletion (2, 32), MutationTaster and PROVEAN (23) were previously used to assess *CXCR4* variants. Additional *in silico* approaches to predict pathogenicity of missense variants include Polyphen-2 and SIFT, but these tools may be of limited use to evaluate *CXCR4* ^WHIM^ variants that primarily comprise nonsense and frameshift variants (35). Indeed, *in silico* algorithms are generally not well suited to predicting pathogenicity of nonsense and frameshift variants. Most *CXCR4* ^WHIM^ variants generate a premature stop codon; therefore, it is important to evaluate whether the variant in question will likely escape nonsense-mediated decay and lead to production of pathogenic protein (28, 29).

Per ACMG-AMP guidelines, only variants classified as pathogenic or likely pathogenic should be used to establish a positive molecular diagnosis (29). Identification of a novel variant of unknown significance poses a significant challenge in clinical decision-making (36). In cases where the clinical data, family history, variant frequency, and/or *in silico* modeling data are not sufficient for a confident variant interpretation, functional biochemical studies can serve as a powerful tool for obtaining additional evidence of pathogenicity (28, 29, 35, 37, 38). The particulars of CXCR4-specific functional tests will be discussed in the next section.

Per ACMG-AMP guidelines, clinical laboratories should implement an internal system to track sequence variants, evidence assertions, and variant classifications (29). Invitae, a certified clinical diagnostic laboratory and the largest submitter of variant data to ClinVar, performed *CXCR4* variant interpretation according to Sherloc framework (28), a refined version of the ACMG-AMP criteria (29), considering all applicable lines of evidence. Thirty of the 36 previously observed *CXCR4* ^WHIM^ variants have been classified as pathogenic, and 6 variants as likely pathogenic for WHIM syndrome based on public databases, clinical data at Invitae, published literature at the time of observation, and functional studies. Absence or low frequency in the general population (per gnomAD), segregation with disease, *de novo* occurrence in affected individual, reports of multiple unrelated cases, variant type (frameshift, nonsense, missense), expected consequence for the gene product (disruption of C-terminus, escape nonsense-mediated decay) and experimental data (impaired internalization) were factors that conferred pathogenic points for CXCR4 variant classification (**Supplementary Table 1**) (8).

## 4 Functional testing for variant interpretation

CXCR4 is a 7-transmembrane G protein-coupled receptor that binds cognate ligand C-X-C motif chemokine ligand 12 (CXCL12)/stromal cell-derived factor 1 (39, 40) to regulate leukocyte trafficking and B-cell development (32, 41–43). The cytoplasmic C-terminal tail CXCR4 harbors a set of phosphorylation motifs that regulate downstream signaling, ß-arrestin binding and internalization of the receptor (44, 45). At the molecular level, CXCR4^WHIM^ variants eliminate or dysregulate the C-terminal phosphorylation, resulting in impaired CXCL12-induced receptor internalization (21), leading to hyperactive (gain-of-function) signaling to downstream pathways (5, 21, 46). These altered responses to CXCL12 underlie the clinical manifestations of WHIM syndrome including enhanced leukocyte retention in bone marrow and defects in adaptive immunity (reviewed in Heusinkveld and Majumdar (3, 47)).

**Figure 2:**
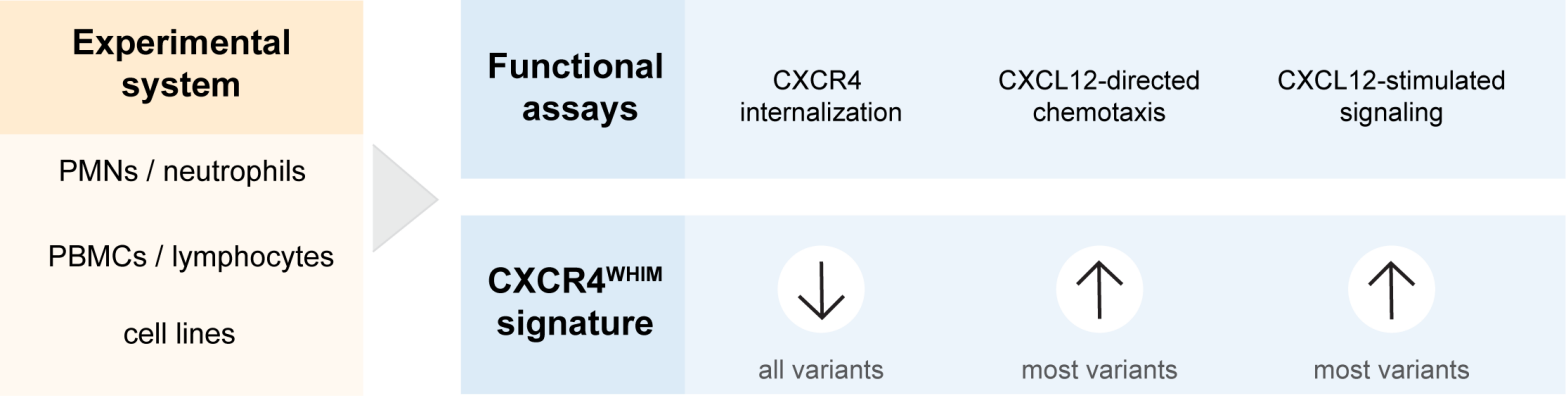
Pipeline of functional tests to support CXCR4 variant interpretation. CXCL12, C-X-C chemokine ligand 12; CXCR4, C-X-C chemokine receptor 4; PBMC, peripheral blood mononuclear cell; PMN, polymorphonuclear leukocyte.

These defects in leukocytes from individuals with WHIM syndrome can be utilized as evidence for a deleterious effect associated with newly identified CXCR4 variants (**Figure 2**). Most previously published studies have used assays to measure CXCR4 internalization in response to CXCL12 stimulation to show the altered function of CXCR4^WHIM^ in comparison with CXCR4^WT^-expressing cells (2, 13–15, 21, 46, 48, 49). The decrease of internalization is the most consistent defect among the entire spectrum of CXCR4^WHIM^ variants. Cellular chemotaxis in response to CXCL12 (12–14, 21, 46, 48, 49), intracellular calcium mobilization (5, 12–15, 22, 46, 49), and PI3K-Akt/extracellular signal-regulated kinase activation assays (13, 22, 46, 49) have also been frequently used, but the gain-of-function phenotypes in functional assays have not been uniform across all variants, with early frameshift variants lacking the hyperactive phenotype (13, 49). Additional assays used to investigate the molecular pathology downstream of CXCR4^WHIM^ receptor comprise β-arrestin recruitment (impaired in CXCR4^WHIM^-expressing cells) (13, 22), G_i_-protein dissociation (increased) (13), reduction in intracellular cyclic adenosine monophosphate level (increased or equal) (13, 49), and F-actin polymerization (increased) (21, 48). To probe the potential pathogenicity of a newly discovered variant, we recommend assessing CXCR4 internalization in response to a range of CXCL12 concentrations, and chemotaxis or downstream signaling assays to test for gain-of-function phenotype.

CXCR4 is expressed on the cell surface of mature leukocytes and their progenitors (50), in addition to other cell types such as endothelial cells; therefore, functional experiments can be performed directly with various types of leukocytes isolated from patient blood (**Figure 2**). Polymorphonuclear leukocytes (12, 14, 46) and peripheral blood mononuclear cells (14, 15, 21, 46) have been used previously to investigate the cellular impairment in CXCR4^WHIM^ variants. However, polymorphonuclear leukocytes may not be preferred due to their short life span and infeasible cryopreservation. Use of peripheral blood mononuclear cells overcomes these drawbacks and additionally enables expansion of T-cell lymphoblasts (12, 13) or generation of immortalized B-cell lines (5, 48) when larger numbers of cells are needed for CXCR4^WHIM^ cellular analysis. Furthermore, cell lines transfected with *CXCR4* variants of interest can be used to decipher the pathobiology downstream of CXCR4 receptor in parallel to functional studies with patient peripheral blood mononuclear cells or as an alternative when patient samples are not available (**Figure 2**). This approach also allows the study of CXCR4 variants in a more standardized and isolated experimental system as they are presented in a homogenous genetic background, allowing direct comparisons of a larger array of variants. Cellular models relying on the overexpression of variant CXCR4 include K562 (2, 14, 15, 46, 49), HEK293 (13, 15, 21, 22), CHO-K1 (21, 46), A0.01 T-cell (21), and Jurkat cell lines (51). In addition, there has been a report of using CRISPR/Cas9 gene editing system to introduce the c.1000C>T p. R334* variant in the endogenous *CXCR4* locus in the Jurkat cell line to overcome some of the concerns with the overexpression systems (49).

## 5 WHIM syndrome: beyond *CXCR4* variants

A small proportion of WHIM syndrome cases were found to be not linked to variants in *CXCR4*. Two unrelated individuals with a clinical diagnosis of WHIM syndrome, but negative for pathogenic *CXCR4* variants, were reported by Balabanian et al. (21). These patients had dysfunction in GRK3, a kinase involved in CXCR4 C-tail phosphorylation (45), resulting in impairment of CXCR4 internalization and thus phenocopying the effects of C-terminal CXCR4 truncation in functional assays (21, 52). Severe congenital neutropenia with myelokathexis and recurrent infections can be also caused by deficiency in CXCR2, a chemokine receptor that mediates neutrophil egression from the bone marrow (53, 54). Mechanistically, CXCR2 loss-of-function recapitulates the pathogenic mechanism of CXCR4 gain-of-function in neutrophils, specifically their excessive retention in bone marrow and impaired egress (55). In terms of functional assays, the 2 published studies on CXCR2 utilized chemotaxis assays showing an impaired chemotactic response to CXCL8 in cells harboring the variant CXCR2 receptors. Although OMIM lists the disease as WHIM syndrome 2 (#619407), it remains to be determined whether these patients fall within the clinical spectrum of WHIM syndrome, as lymphopenia, hypogammaglobulinemia, and warts have not been reported in this patient group thus far (54).

## 6 Conclusion and perspectives

WHIM syndrome is a clinicopathologic diagnosis, and since the initial discovery of the disease, our understanding of its natural history and spectrum of clinical manifestations continues to evolve, and diagnosis of WHIM syndrome remains challenging (2, 3). Increased implementation of genetic testing can expedite and support the clinical diagnosis of WHIM syndrome but relies on annotation of likely pathogenic variants (29). The catalogue of disease-causing CXCR4^WHIM^ variants has grown to 36 (8), and additional novel variants in the C-terminus of the receptor are likely to be detected in the future. The current body of evidence, including patient observations and functional studies, is large enough to make a prediction that any novel truncating variant (nonsense or frameshift) between aa 317 and 346 will likely be a pathogenic variant for WHIM syndrome. A similar prediction for missense variants is not possible, and such variants will have to be assessed for evidence of pathogenicity on an individual basis using cellular functional assays. Of interest is whether activating and/or non-”desensitizable” variants outside of the currently curated C-terminal “hot spot” for CXCR4^WHIM^ variants will be found in individuals with WHIM syndrome. In these instances, a rigorous variant interpretation should be done according to ACMG-AMP guidelines, which will require integrating clinical and functional data. With respect to functional tests, impaired C-X-C chemokine ligand 12−induced CXCR4 internalization has been most consistently associated with pathogenic CXCR4^WHIM^ variants, and it seems to correlate with a decrease in circulating neutrophils in individuals with WHIM syndrome (49). In addition, the genetic landscape of WHIM syndrome may not be restricted to *CXCR4*; defects in other genes regulating either CXCR4 trafficking or the balance between neutrophil mobilization and retention in bone marrow lead to a spectrum of WHIM-like disease presentations (21, 48, 53, 54, 56). Increased implementation of whole genome/exome sequencing in the diagnostic process of primary immunodeficiencies warrants new discoveries in the future. Understanding the genotypic-phenotypic associations in WHIM syndrome has the potential to improve time to diagnosis and guide appropriate clinical management resulting in a true example of precision medicine. Thus far, there is no standard-of-care treatment addressing the underlying cause of WHIM syndrome resulting in use of therapies focused only on clinical signs and symptoms (57). Therefore, efforts are focused on development of therapies targeting the underlying dysfunction in CXCR4 signaling pathways. Recently, an orally bioavailable small-molecule CXCR4 antagonist, mavorixafor, was approved by the US Food and Drug Administration for the treatment of patients with WHIM syndrome (58).

## Author contributions

KZ: Conceptualization, Data curation, Formal analysis, Investigation, Methodology, Visualization, Writing – original draft, Writing – review & editing. KN: Writing – original draft, Writing – review & editing. GB: Writing – original draft, Writing – review & editing. MY: Writing – review & editing. JW: Writing – original draft, Writing – review & editing.
