# Supplementary material for: Expanding CXCR4 variant landscape in WHIM syndrome: integrating clinical and functional data for variant interpretation
Source: Front Immunol. 2024 Jul 8;15:1411141. doi: 10.3389/fimmu.2024.1411141 (PMC11260667; doi:10.3389/fimmu.2024.1411141)
Supplement: Supplementary file 1 [file Table_1.docx]

Supplementary Material

**Supplementary Table 1.** CXCR4 variants identified in individuals with WHIM syndrome and the variants’ current interpretation in ClinVar, in Geier et al 2022, and based on the ACMG-AMP/Sherloc guidelines. ACMG-AMP, American College of Medical Genetics and the Association for Molecular Pathology; c, coding DNA sequence; FS, frameshift; HGVS, Human Genome Variation Society; LP, likely pathogenic; MS, missense; NS, nonsense; P, pathogenic; p, protein; VUS, variant of uncertain significance; $, functional *in vitro* evidence available for the variant.

| **HGVS c-name** | **HGVS p-name** | **Variant type** | **Reference** | **ClinVar interpretation** | **Geier et al interpretation** | **Sherloc interpretation** |
| --- | --- | --- | --- | --- | --- | --- |
| c.1027G>A | p.Glu343Lys | MS | PMID: 22596258 | - | - | LP^$^ |
| c.994 G>T | p.Gly332* | NS | ClinVar variation ID: 574352 | P | - | P^$^ |
| c.997A>T | p.Lys333* | NS | PMID: 24139496 | - | - | LP^$^ |
| c.1000C>T | p.Arg334* | NS | PMID: 12692554 | P | P | P^$^ |
| c.1006G>T | p.Gly336* | NS | PMID: 15026312 | P | - | P^$^ |
| c.1013C>G | p.Ser338* | NS | PMID: 15536153 | P | P | P^$^ |
| c.1013C>A | p.Ser338* | NS | PMID: 35947323 | LP | LP | P |
| c.1027G>T | p.Glu343* | NS | PMID: 12692554 | P | LP | P^$^ |
| c.1037_1040del | p.Ser346* | NS | PMID: 35947323 | - | LP | P^$^ |
| c.950_953del | p.Leu317Profs*3 | FS | PMID: 32499645 | P/LP | - | P^$^ |
| c.951del | p.Thr318Pro fs*3 | FS | PMID: 36883568 | - | - | P^$^ |
| c.952dup | p.Thr318Asnfs*26 | FS | PMID: 35947323 | P | LP | LP^$^ |
| c.954del | p.Ser319Leufs*2 | FS | PMID: 35947323 | - | VUS | P^$^ |
| c.956_957del | p.Ser319Cysfs*24 fs*24 | FS | PMID: 27484033 | LP | - | P^$^ |
| c.959_960del | p.Val320Glufs*23 | FS | PMID: 31942606 | P | - | P^$^ |
| c.963dup | p.Arg322Glnfs*22 | FS | PMID: 35947323 | VUS | LP | P^$^ |
| c.964dup | p.Arg322Lysfs*22 | FS | Invitae | - | - | LP |
| c.966_967del | p.Gly323Val fs*20 | FS | PMID: 28643496 | - | - | P^$^ |
| c.969dup | p.Ser324Val fs*20 | FS | PMID: 23009155 | VUS | - | P^$^ |
| c.970_971insTCCT | p.Ser324Phefs*21 | FS | PMID: 35947323 | - | LP | P^$^ |
| c.970del | p.Ser324Profs*42 | FS | PMID: 32870250 | - | - | P^$^ |
| c.969del | p.Ser324Profs*42 | FS | PMID: 35947323 | - | LP | P^$^ |
| c.976dup | p.Leu326Profs*18 | FS | PMID: 35947323 | - | LP | P^$^ |
| c.977_978del | p.Leu326Glnfs*17 | FS | PMID: 35947323 | - | LP | P^$^ |
| c.979_980insG | p.Lys327Argfs*17 | FS | PMID: 35947323 | - | LP | P^$^ |
| c.986_990del | p.Leu329Glnfs*13 fs*13 | FS | PMID: 27059040 | - | - | P^$^ |
| c.988_989del | p.Ser330Glnfs*13 | FS | ClinVar variation ID: 1163801 | P | - | LP^$^ |
| c.1016_1017del | p.Ser339Cys fs*4 | FS | PMID: 12692554 | P | - | P^$^ |
| C.1012_1015dup | p.Ser339Phe fs*6 | FS | PMID: 34973340 | P | LP | P^$^ |
| c.1014del | p.Ser339Leufs*27 | FS | PMID: 35947323 | VUS | VUS | P^$^ |
| c.1016_1017dup | p.Val340Leufs*27 | FS | PMID: 35947323 | - | LP | P^$^ |
| c.1021del | p.Ser341Profs*25 | FS | PMID: 19321197 | - | - | P^$^ |
| c.1025_1028del | p.Thr342Ser fs*23 *23 | FS | ClinVar variation ID: 1319371 | LP | - | P^$^ |
| c.1025_1026del | p.Thr342Argfs*3 | FS | ClinVar variation ID: 1494228 | VUS | - | LP^$^ |
| c.1032_1033del | p.Glu345Valfs*12 | FS | PMID: 35493524 | - | - | P |
| c.893_1034dup | p.Ser346Profs*12 | FS | PMID: 35947323 | P | LP | P^$^ |
